# Clonidine Improved Laboratory-Measured Decision-Making Performance in Abstinent Heroin Addicts

**DOI:** 10.1371/journal.pone.0029084

**Published:** 2012-01-23

**Authors:** Xiao-Li Zhang, Gui-Bin Wang, Li-Yan Zhao, Li-Li Sun, Jun Wang, Ping Wu, Lin Lu, Jie Shi

**Affiliations:** National Institute on Drug Dependence, Peking University, Beijing, China; Baylor College of Medicine, United States of America

## Abstract

**Background:**

Impulsivity refers to a wide spectrum of actions characterized by quick and nonplanned reactions to external and internal stimuli, without taking into account the possible negative consequences for the individual or others, and decision-making is one of the biologically dissociated impulsive behaviors. Changes in impulsivity may be associated with norepinephrine. Various populations of drug addicts all performed impulsive decision making, which is a key risk factor in drug dependence and relapse. The present study investigated the effects of clonidine, which decreased norepinephrine release through presynaptic alpha-2 receptor activation, on the impaired decision-making performance in abstinent heroin addicts.

**Methodology/Principal Findings:**

Decision-making performance was assessed using the original version of Iowa Gambling Task (IGT). Both heroin addicts and normal controls were randomly assigned to three groups receiving clonidine, 0, 75 µg or 150 µg orally under double blind conditions. Psychiatric symptoms, including anxiety, depression and impulsivity, were rated on standardized scales. Heroin addicts reported higher scores on the Barratt Impulsiveness Scale and exhibited impaired decision-making on the IGT. A single high-dose of clonidine improved the decision-making performance in heroin addicts.

**Conclusions/Significance:**

Our results suggest clonidine may have a potential therapeutic role in heroin addicts by improving the impaired impulsive decision-making. The current findings have important implications for behavioral and pharmacological interventions targeting decision-making in heroin addiction.

## Introduction

The high propensity to relapse is a hallmark feature of drug addiction and a main challenge for treatment strategies. Impulsivity, which refers to behavior performed with little or inadequate forethought, is a key vulnerability factor in drug addiction and risk factor in relapse [1]. Chronic drug abusers showed elevated scores on questionnaires measures of impulsivity [2] and impaired performance on laboratory tasks of impulsivity [3], [4].

Impulsivity is a multifactorial construct that can be measured by tasks such as decision-making, response inhibition, and reflective impulsivity [5], [6]. The Iowa Gambling Task (IGT) [7] was specifically developed to assess and quantify the decision-making ability by simulating the real-life decision under ambiguous conditions [8]. Converging evidence indicates that decision-making performance is impaired in subjects abusing opiate [9], [10], alcohol [11], [12], psychostimulants [11], [13], and marijuana [14].

Prefrontal cortical (PFC) network plays an important role in gating impulsive actions in a variety of behavior tasks [15], [16]. Activation of alpha-1 noradrenergic receptor is associated with short-term changes in PFC function that are consistent with increased impulsivity [17]. Research in normal subjects found that laboratory-measured impulsivity was increased by acute administration of yohimbine, which augments norepinephrine release by blocking alpha-2 noradrenergic receptors [18]. Our recent study has found that the decision-making was impaired in former heroin addicts up to abstinence for 2 years, and stress can exacerbate an already existing impairment of decision-making, or unmask a latent one [19]. Clonidine may decrease norepinephrine release through presynaptic alpha-2 receptor activation, resulting in decreased levels of norepinephrine metabolites [20]. However, there has been no research about the effects of clonidine on decision-making performance in abstinent heroin addicts.

Thus, our hypothesis was that, at doses that decreased behavioral arousal or activation, clonidine would improve decision-making performance in abstinent heroin addicts. Since 150 µg clonidine can suppress blood pressure [21], [22], we also examined the safety and efficacy of this medication at half of the dose (e.g. 75 µg) in abstinent heroin addicts and hypothesized that the effect of clinidine may be dose-dependent.

## Results

Fifty-three heroin addicts and 56 normal controls were enrolled and completed the study. The demographic, substance abuse characteristics, as well as BDI and HAMA scores of the participants were summarized in Table 1. One-way ANOVA indicated a significant effect of Group on self-report ratings of depression on the BDI-II (*F*_(5,108)_ = 16.38, *p*<0.05). The analysis revealed that each subgroup of heroin addicts scored higher on depression compared with controls (*p*<0.05). No significant differences in age, education and anxiety score were found in the three subgroups of heroin addicts and normal controls. No significant differences were found in drug use characteristics (duration of heroin use and average dose abused) across heroin groups.

**Table 1:** Demographic and clinical characteristics of participants.

Characteristic	Control groups			Heroin groups		
	*Placebo (n = 20)*	*75 µg clonidine (n = 18)*	*150 µg clonidine (n = 18)*	*Placebo (n = 17)*	*75 µg clonidine (n = 18)*	*150 µg clonidine (n = 18)*
Age (years)	29.20(4.72)	31.33 (7.20)	29.83 (5.83)	32.65 (6.71)	32.11 (5.86)	30.5 (6.29)
Education (years)	11.10(2.40)	10.50 (2.12)	11.94 (2.36)	10.06 (2.38)	10.06 (2.34)	9.83 (2.20)
Duration of heroin use (years)				7.92 (3.45)	7.72 (4.60)	7.00 (4.65)
Dose used (g/d)				0.89 (0.46)	0.85 (0.67)	0.83 (0.45)
Abstinence time (months)				5.39 (2.36)	5.38 (2.35)	5.20 (2.90)
HAMA score	2.80 (1.67)	2.22 (1.63)	2.56 (3.07)	3.71 (1.76)	3.17 (1.65)	3.00 (0.91)
BDI-II score^a^	3.10 (3.80)	2.50 (3.20)	3.83 (4.55)	12.9 (5.32)	9.67 (5.11)	10.78 (5.99)

Values are expressed as mean (S.D.). No significant differences were found in age, education, and HAMA score across groups. No significant differences were found for heroin abuse characteristics (duration, dose, and abstinence time).

a Difference between heroin addicts and normal controls.

### Heroin addicts reported higher impulsivity scores on the BIS-11

**Table 2** showed self-reported impulsivity on the BIS-11 in heroin addicts and normal controls. The analysis revealed a significant effect of Group (*F*_(1,108)_ = 54.288, *p*<0.05) on self-report ratings of the BIS-II total score, which meant that heroin addicts reported higher scores than normal controls. Significant differences were also found on the subscales motor impulsiveness (*F*_(1,108)_ = 17.789, *p*<0.05), attention impulsiveness (*F*_(1,108)_ = 24.77, *p*<0.05), and nonplanning impulsiveness (*F*_(1,108)_ = 47.254, *p*<0.05). No significant differences were found between the three subgroups of heroin addicts and normal controls in total scores and subscale scores of self-reported impulsivity on the BIS-II (*p*>0.05).

**Table 2:** Self-reported impulsivity on the BIS-II in heroin addicts and normal controls.

Scale/subscale	Control groups			Heroin groups		
	*Placebo (n = 20)*	*75 µg clonidine (n = 18)*	*150 µg clonidine (n = 18)*	*Placebo (n = 17)*	*75 µg clonidine (n = 18)*	*150 µg clonidine (n = 18)*
BIS: Attention^a^	14.90 (3.24)	14.56 (3.76)	15.33 (3.87)	17.18 (2.70)	17.78 (2.10)	18.67 (2.50)
BIS: Motor^a^	21.35 (3.76)	22.22 (3.66)	21.06 (4.33)	25.00 (3.14)	24.28 (2.52)	23.83 (3.24)
BIS: Nonplanning^a^	21.60 (4.90)	23.39 (4.22)	22.89 (4.42)	28.88 (4.81)	28.28 (4.42)	28.17 (3.54)
BIS: Total^a^	58.25(10.29)	60.17 (7.33)	59.28 (8.45)	71.77 (8.53)	70.33 (6.86)	70.67 (6.62)

Values are expressed as mean (S.D.).

a Difference between heroin addicts and normal controls.

### 150 µg clonidine improved the impaired decision-making in heroin addicts

**Figure 1:**
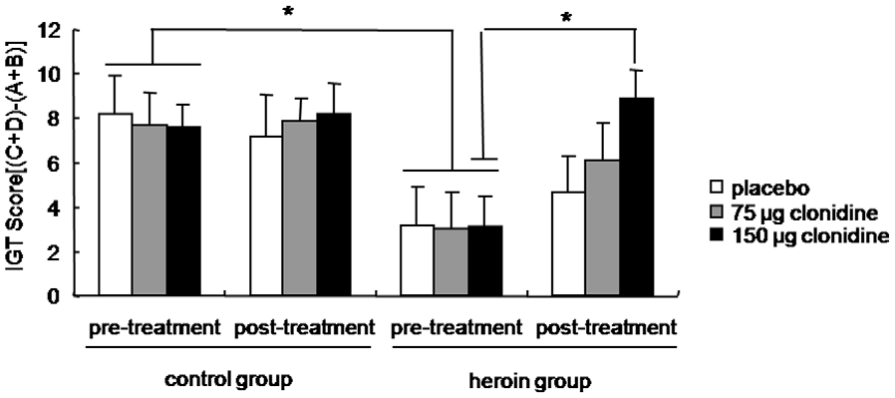
Effects of clonidine on the Iowa Gambling Task in heroin addicts assessed using the original version of IGT net scores *[(C+D)−(A+B)]*. Data are expressed as mean ± SEM. Positive net scores reflect advantageous (non-impaired performance), while negative net scores reflect disadvantageous (impaired performance). A significant difference was found between heroin addicts and normal controls. A dose of 150 µg clonidine significantly increased IGT net scores in heroin addicts. *p<0.05, significant difference from placebo subgroup in *post hoc t*-test.

Fig. 1 showed the change of IGT overall net scores *[(C+D)−(A+B)]* in heroin addicts and normal controls. The analysis revealed that the heroin group tended to select more disadvantageous cards in the IGT at baseline compared with the control group (*F*_(1,108)_ = 6.09, *p*<0.05). No difference was found across the three subgroups in both the control group and heroin group (*p*>0.05). The analysis revealed a significant effect of Time (*F*_(1,102)_ = 4.495, *p*<0.05), a Time×Group interaction (*F*_(1,102)_ = 4.687, *p*<0.05), and a Time×Dose interaction (*F*_(2,102)_ = 2.24, *p*<0.05). In the heroin addict groups, two-way ANOVA revealed a significant effect of Time (*F*_(1,49)_ = 8.17, *p*<0.05) and a Time×Dose interaction (*F*_(2,49)_ = 2.68, *p*<0.05). In within-group comparisons, 150 µg clonidine, but not 75 µg clonidine or placebo, improved decision-making performance (*p*<0.05). In all three subgroups of normal controls, no significant difference was found between pretreatment and post-treatment (*p*>0.05).

### 150 µg clonidine decreased blood pressure of heroin addicts

**Figure 2:**
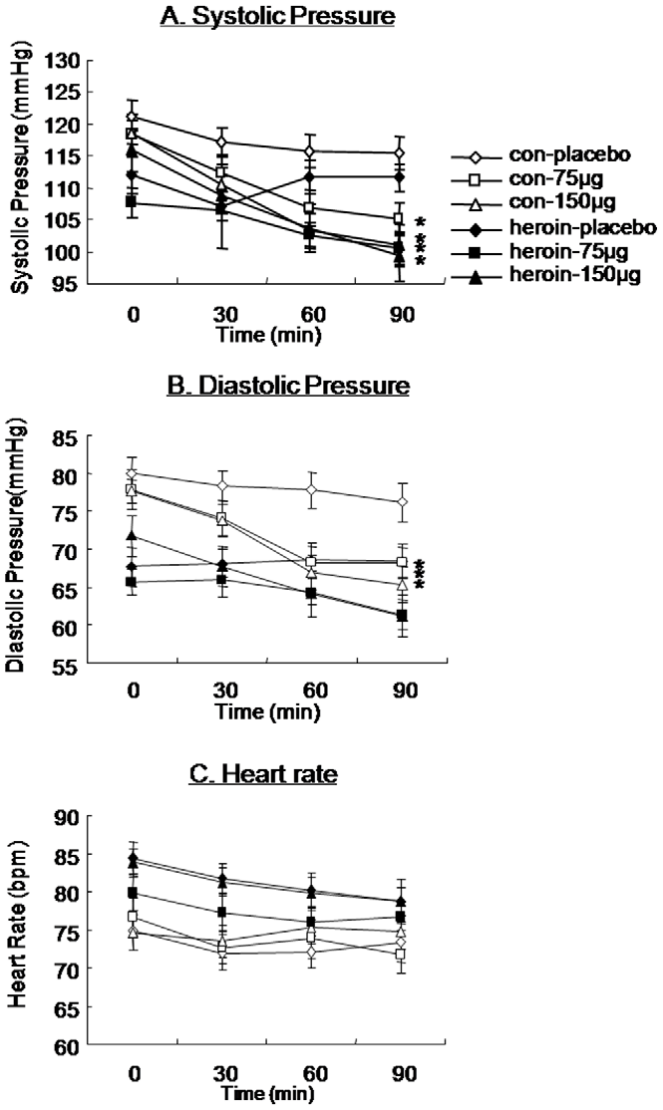
Effects of clonidine on blood pressure and heart rate in heroin addicts. (A) Systolic blood pressure. Treatment with 75 µg and 150 µg clonidine significantly decreased systolic blood pressure at 90 min in both heroin addicts and normal controls. (B) Diastolic blood pressure. Treatment with 150 µg clonidine significantly decreased diastolic blood pressure at 90 min in both heroin addicts and normal controls. Treatment with 75 µg clonidine significantly decreased diastolic blood pressure at 90 min in normal controls. (C) Heart rate. No effect of clonidine was found on heart rate. *p<0.05, compared with baseline in post hoc t-test. Data are expressed as mean ± SEM.

Fig. 2 showed changes in systolic blood pressure (SBP), diastolic blood pressure (DBP) and heart rate (HR) after clonidine or placebo treatment in the heroin and control groups. There was no significant difference in baseline SBP, DBP, or HR between groups (*p*>0.05). Both doses of clonidine decreased SBP of heroin addicts and control subjects (Time effct: (*F*_(3,306)_ = 40.01, *p*<0.05; Time×Dose interaction: (*F*_(6,306)_ = 7.17, *p*<0.05) (Fig. 2A), but not heart rate (*p*>0.05; Fig. 2C). 150 µg clonidine significantly decreased DBP in both control and heroin groups, but 75 µg clonidine only reduced DBP in the control group (Time effect: (*F*_(3,309)_ = 42.07, *p*<0.05), Time×Group interaction (*F*_(3,309)_ = 5.09, *p*<0.05), Time×Dose interaction (*F*_(6,309)_ = 8.77, *p*<0.05)) (Fig. 2B).

## Discussion

**The present placebo-controlled, double-blind study demonstrated that 150 µg clonidine improved the impaired decision-making performance in abstinent heroin addicts, which was assessed with the original card version of the IGT.** The impulsive decision-making in heroin addicts were consistent with previous studies in opiate dependents using IGT, Game of Dice Task [23] and Cambridge Gambling Task [24], [25]. **The lower dose of 75 µg clonidine did not show the significant effect on the decision-making ability in heroin abusers. But the pharmacokinetics study has found the cumulative concentration of chronic dosing with 75 µg clonidine** [**26**] **, which suggested this lower dose of clonidine could be effective to enhance the decision-making ability.**

Studies using neuroimaging and brain lesion technologies have implicated that prefrontal cortex (PFC), medial frontal cortex and basal ganglia in controlling impulsive decision making [27]. Various neurotransmitter systems, including norepinephrine, dopamine and serotonin, in the above brain regions modulated neural processes underlying the decision-making [28]. Decreased activation in the specific PFC areas have been found in addicts [13], [29], [30].

It has been suggested that stimulation of the alpha-2 adrenoceptor improved PFC function in animals [31], [32]. For example, intra-PFC infusion of the alpha-2 agonist guanfacine in aged monkeys [20] or rats [33] improved working memory performance. Conversely, infusion of the alpha-2 antagonist yohimbine into the PFC in monkeys impaired working memory performance [34]. Moreover, acute yohimbine increased laboratory-measured impulsivity [18], [35]. In the present study, the improvement of decision-making performance in abstinent heroin addicts by 150 µg clonidine may attribute to presynaptic alpha-2 receptor activation and the subsequent decreased norepinephrine release in the PFC.

Some limitations of our present study should be noted. First, retest of IGT may produce a practice effect. However, the decision-making capacity of drug addicts is similar to that of patients with ventromedial prefrontal cortex (vmPFC) damage [36], who did not improve their performance on the gambling task when tested repeatedly [37]. Moreover, abnormalities in the vmPFC region were found in cocaine addicts [38]. **Second, our present results did not show the low dose of clonidine significantly improved the decision-making ability, which may attribute to the small sample size. The further research work should enlarge the number of subjects.** The third potential limitation is that the noradrenergic effects of clonidine were not measured directly in this preliminary study. However, both the 75 µg and 150 µg clonidine decreased blood pressure significantly. It has been shown that clonidine decreased the norepinephrine metabolites and altered working memory performance [39], [40] at doses similar to those used here. Clonidine could infulence other transmitter systems through alpha-2 receptors [41], [42]. Routine monitoring of blood pressure was necessary to confirm safety in heroin addicts in future study because of the significant reduction of blood pressure by clonidine.

In conclusion, we found that abstinent heroin addicts perform more impulsively, and clonidine may play a potential therapeutic role in improving the impaired decision-making performance. These findings have important implications for behavioral and pharmacological interventions targeting impulsivity in heroin addiction.

## Materials and Methods

The study was approved by the Peking University Research Ethics Board. All participants provided written informed consent and received monetary compensation for taking part in the study.

### Participants

Eighty-six male abstinent heroin addicts were recruited from the Yichang Compulsory Rehabilitation Center, Hubei province, China. Sixty-one normal subjects were recruited through advertisements followed by telephone screening. All subjects were 20 to 40 years old. The study's procedures and risks were explained and discussed thoroughly, and written informed consent was obtained from subjects before study participation. Prior to enrollment, the addicts were screened using the Structured Clinical Interview for the *Diagnostic and Statistical Manual of Mental Disorders*, 4th edition (DSM-IV) [43] and underwent a thorough interview about their medical history and a physical examination (electrocardiogram, blood pressure, heart rate). Exclusion criteria included a history of head injury or a self-reported history of severe mental illness or psychosis neurological disorders. Heroin addicts needed to be drug free for at least 1 month with no current medical illness requiring pharmacologic treatment or any other substance abuse with the exception of heroin and nicotine (assessed by urine drug screen and self-report). They have been treated with methadone for one week, and the dose regimen was established basing on their body weight, the doses they took before and severity of withdrawal syndrome, etc.

### Questionnaires and neuropsychological measures

#### Demographic characteristics

Demographic characteristics of the subjects were collected upon arrival, including gender, age, education years, heroin use time and dose, time of last drug use, and smoking status.

#### Hamilton Anxiety Scale

The 14-item Hamilton Anxiety Scale (HAMA) questionnaire was used to measure anxiety. Items 1–6 and 14 were about psychological anxiety, and items 7–13 were about somatic anxiety [44].

#### Beck Depression Inventory

Depressive symptoms were measured using the 13-item Beck Depression Inventory (BDI). Each of the 13 items measured the presence and severity of depressive symptoms by requiring a self-rating from 0 to 3 [45].

#### Barratt Impulsiveness Scale version-11

The Barratt Impulsiveness Scale (BIS-11) is a validated questionnaire that measures different dimensions of impulsivity [46]. The questionnaire is a 30-item self-report instrument that contains three subscales: motor impulsiveness (e.g., I am restless at the theatre or lectures), attention impulsiveness (e.g., I get easily bored when solving thought problems), and nonplanning impulsiveness (e.g., I spend or charge more than I earn). The Chinese version of the BIS-11 has been shown to have good internal consistency and test-retest reliability [47], [48].

#### Iowa Gambling Task

We used the original card version of the IGT to evaluate decision-making performance. The task was administered according to the procedure described by the Iowa group [7], [49]. In the task, the disadvantageous decks of cards yield higher immediate rewards but unpredictable and larger delayed punishments, while the advantageous decks provide lower immediate gains but unexpected and smaller future losses. Quality of decision-making was measured by the overall net score, which is obtained by subtracting the number of cards selected from the disadvantageous decks from the advantageous decks *[(C+D)−(A+B)]*.

### Blood pressure and heart rate assessment

Systolic and diastolic blood pressure was measured with a 9062D monitor (Baozhong Biotechnology Company, Beijing, China). A pulse sensor was attached to the subject's finger and connected to an SD-700 monitor to provide a continuous measure of pulse [50]. Multiple measures of pulse and blood pressure were monitored for baseline and every 30 min after treatment.

### Procedure

Following recruitment, both the normal participants and heroin addicts were randomly assigned to three groups: placebo, 75 µg and 150 µg clonidine (Changzhou Pharmaceuticals, Jiangsu, China) group. The participants and the experimenters were blind to the study treatment. Each participant attended a testing session at the clinical laboratory at 8:00 am. Upon arrival, the experimental procedure was explained to each participant (Table 3). Following a 15 min adaptation time in which subjects were instructed to relax, each participant completed the questionnaires (HAMA, BDI, BIS) and neuropsychological test (IGT). After baseline testing of blood pressure and heart rate, subjects received placebo, 75 µg clonidine, or 150 µg clonidine orally. Clonidine doses were based on previous studies [51], [52]. The decision-making test was repeated 90 min later. Blood pressure and heart rate were monitored every 30 min. Participants were allowed to leave the laboratory when their systolic blood pressure and heart rate returned to baseline levels.

**Table 3:** Schedule for laboratory session.

Time	Event/activity
*8:00 AM*	Participant arrival, written informed consent given
*8:15 AM*	Demographic characteristic collection, HAMA, BDI, BIS questionnaire, IGT, online baseline heart rate and blood pressure
*8:45 AM*	Clonidine/placebo administration, online baseline heart rate and blood pressure
*9:15 AM*	Online baseline heart rate and blood pressure
*9:45 AM*	Online baseline heart rate and blood pressure
*10:30 AM*	IGT
*10:45 AM*	10 min relaxation time

### Statistical analysis

All data were analyzed with SPSS v. 13.0 (Chicago, IL, USA). Demographic characteristics (i.e., age, years of education, duration of heroin use, dose used, and duration of abstinence) and psychiatric symptoms, including anxiety, depression, and impulsivity among groups were compared using one-way analysis of variance (ANOVA). Iowa Gambling Task scores were analyzed by three-way ANOVA with the between-subjects factors of Group (heroin group *vs.* control group) and Dose (placebo, 75 µg, and 150 µg clonidine) and the within-subjects factor of Time (pretreatment vs. post-treatment). In the heroin addict groups, IGT scores were analyzed by two-way ANOVA with the between-subjects factor of Dose (placebo, 75 µg, and 150 µg clonidine) and the within-subjects factor of Time (pretreatment vs. post-treatment). Repeated-measures ANOVA with the between-subjects factors of Group (heroin group *vs.* control group) and Dose (placebo, 75 µg, and 150 µg clonidine) and the within-subjects factor of Time (0, 30, 60, and 90 min) were used to analyze systolic blood pressure, diastolic blood pressure and heart rate. *Post hoc* pairwise comparisons were conducted with Fisher's Least Significant Difference (LSD) test. Values of *p*<0.05 were considered statistically significant. Means are given with standard deviations.
